# Exercise quantification from single camera view markerless 3D pose estimation

**DOI:** 10.1016/j.heliyon.2024.e27596

**Published:** 2024-03-12

**Authors:** Clara Mercadal-Baudart, Chao-Jung Liu, Garreth Farrell, Molly Boyne, Jorge González Escribano, Aljosa Smolic, Ciaran Simms

**Affiliations:** a Trinity College Dublin, Ireland; b Leinster Rugby, Ireland; c Lucerne University of Applied Sciences and Arts, Ireland

**Keywords:** Pose estimation, Motion capture, Sports biomechanics, Injury biomechanics, Computer vision, Markerless

## Abstract

Sports physiotherapists and coaches are tasked with evaluating the movement quality of athletes across the spectrum of ability and experience. However, the accuracy of visual observation is low and existing technology outside of expensive lab-based solutions has limited adoption, leading to an unmet need for an efficient and accurate means to measure static and dynamic joint angles during movement, converted to movement metrics useable by practitioners. This paper proposes a set of pose landmarks for computing frequently used joint angles as metrics of interest to sports physiotherapists and coaches in assessing common strength-building human exercise movements. It then proposes a set of rules for computing these metrics for a range of common exercises (single and double drop jumps and counter-movement jumps, deadlifts and various squats) from anatomical key-points detected using video, and evaluates the accuracy of these using a published 3D human pose model trained with ground truth data derived from VICON motion capture of common rehabilitation exercises. Results show a set of mathematically defined metrics which are derived from the chosen pose landmarks, and which are sufficient to compute the metrics for each of the exercises under consideration. Comparison to ground truth data showed that root mean square angle errors were within 10° for all exercises for the following metrics: shin angle, knee varus/valgus and left/right flexion, hip flexion and pelvic tilt, trunk angle, spinal flexion lower/upper/mid and rib flare. Larger errors (though still all within 15°) were observed for shoulder flexion and ASIS asymmetry in some exercises, notably front squats and drop-jumps. In conclusion, the contribution of this paper is that a set of sufficient key-points and associated metrics for exercise assessment from 3D human pose have been uniquely defined. Further, we found generally very good accuracy of the Strided Transformer 3D pose model in predicting these metrics for the chosen set of exercises from a single mobile device camera, when trained on a suitable set of functional exercises recorded using a VICON motion capture system. Future assessment of generalization is needed.

## 1 Introduction

The vision of the American Physical Therapy Association is “optimizing movement to improve the human experience” [1,2]. A key role of a physiotherapist is the assessment and management of movement [3]. In a sporting environment, physiotherapists and coaches are tasked with evaluating the movement quality of athletes across the spectrum of ability and experience. These assessments inform the development of training programmes for athletic performance. Movement analysis also plays a principal role in rehabilitation, by quantifying exercise progression and return-to-play timelines.

Despite the proliferation of sports technology equipment, these decisions are still largely made through subjective visual assessment of athlete movement patterns [4]. Physiotherapists and coaches mostly rely on their understanding of human movement and their clinical experience to identify aberrant movements, and then to design and evaluate intervention effectiveness [1]. Objectively, this requires a measure of static and dynamic joint angles and their progression over time. However, the accuracy of visual observation by physiotherapists is limited to around 12° in low-speed functional activities [1], and from experience is considerably lower otherwise. A recent US review of physical therapists found that less than half of them use video-based motion analysis, citing accuracy and ease-of-use as reasons [5]. As a result, movement assessments are often reported qualitatively in physiotherapy notes, making athlete progression over time more difficult to assess. There is therefore an unmet need for an efficient and accurate means to measure static and dynamic joint angles during movement, converted to movement metrics useable by practitioners.

There is a trade-off between ease-of-use, accuracy and cost in quantifying human movement. Human movement metrics are mostly derived from the absolute angle of a segment or angles between adjacent body segments (e.g. maximum knee angle, anterior pelvic tilt, torso and head alignment). Gold standard three-dimensional measurements (e.g. VICON) rely on marker-based laboratory systems, and they are expensive and time-consuming to apply [6]. Two-dimensional video methods are much more cost- and time-effective, but they are generally also less accurate [7]. Wearable inertial measurement methods can have clinical accuracy (e.g. Refs. [8,9], but these rarely measure whole-body movement and the associated financial and time burdens have so far meant that physiotherapists and coaches continue to mainly rely on “by-eye” observation for whole-body movement assessment. Camera based methods using pose landmarks have the potential to overcome all of these limitations, if sufficient detail and accuracy can be achieved, and they are therefore the focus of this work.

Computer vision advances have led to a proliferation of methods for 2D and 3D (e.g. Refs. [[10], [11], [12]]) human pose estimation (HPE), even using a single (monocular) smartphone video. This has the potential to significantly reduce setup time and costs for exercise quantification [13,14]. Methods using depth sensors (eg. Kinect) initially showed promise ([6,15,16]), but have struggled with widespread adoption, partly due to accuracy and practicalities. Two-dimensional HPE is ubiquitous (e.g. OpenPose [17], Meta's Detectron2), and freely available 3D HPE (e.g. VideoPose [12]/PoseLifter [18]) exists but is generally not directly suitable for rehabilitation/sporting applications. Very recently, BlazePose [11] was applied in physiotherapy exercise classification [19], but the predictive capacity for individual joint angles was not clear. Further a new two-camera approach combining 2D keypoint estimation from OpenPose to lift to 3D yielded good accuracy in assessing mean absolute error in joint angles in a variety of activities, including walking and squatting and jumping [20], although the accuracy of individual metrics of interest to physiotherapists was not presented, and a calibration step is needed to combine the output from the two cameras.

Two-dimensional pose estimation is suitable for joint angle predictions in planar movements [e.g. Refs. [21,22]], however this requires precise camera placement and limits the angles that can be analyzed. Three-dimensional joint angle measures remain much more challenging. A recent monocular 3D approach is promising [23], though the dataset used for training does not specifically relate to exercises. Only knee angle was explicitly evaluated, similar to Ref. [24]. Some high-level metrics for exercise performance [25] and a recent framework for automated exercise assessment have been proposed [26,27], but these have not yet been implemented in practice, and the metrics are not readily useable by practitioners.

**Fig. 1:**
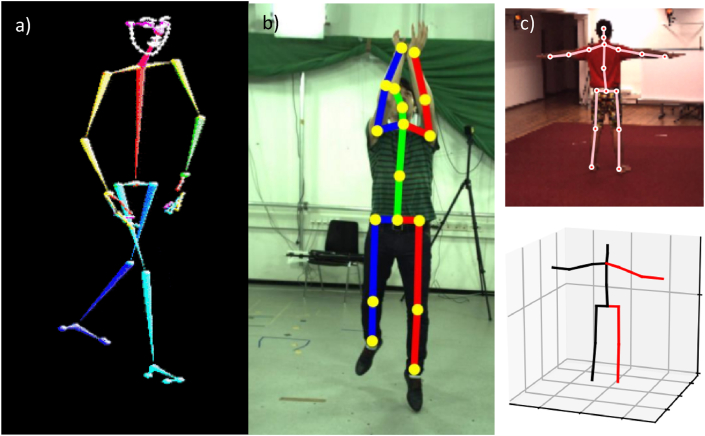
Limitations in existing HPE skeletons biomechanical joint angle computation (examples shown adapted from (a) OpenPose [17], (b) PoseLifter [18] and (c) VideoPose3D [12]. The use of a single key-point for the hips means that orientation of the pelvis cannot be found from these skeleton models, and this in turn limits computation of the actual hip joint angles, and the curvature in the lower spine, amongst other quantities.

Human joints can be mostly modelled as either one (e.g. knee) or three (e.g. hip) degree of freedom rotational joints. To compute a segment orientation, at least three key-points on each segment are required. However, a review of monocular 2D and 3D HPE pose key-points (e.g. Openpose [17], Detectron 2, HRNet [28], VideoPose 3D [12], Poselifter [18]) shows formulations with a single key-point at each hip joint (see Fig. 1). These reduced landmark models are therefore insufficient for use in the calculation of clinically meaningful hip joint angles, which are determined by relative orientation of the pelvis and femur. Similarly, existing 3D HPE skeletons typically assume a highly simplified torso. This simplification is often suitable for gaming and animation, but is inadequate for evaluating pelvic angle and spinal organisation (e.g. anterior pelvic tilt and flexion at different levels of the spine).

The ISB [29] proposed standard conventions for body segment and joint coordinate systems, which have been widely adopted in laboratory and surgical environments (eg VICON) and allow for unambiguous definition of 3D joint angles in research/surgical applications. However, a different method is needed for video-based assessment to quantify joint angles from video in a manner directly interpretable by physiotherapists and coaches in a sporting environment. For this, a suitable set of joint angle metrics computed directly from pose key-points (shoulders/ankles/knees etc) is needed, in a format that physiotherapists and coaches can use directly. The definition of these metrics should correspond as closely as possible to how experienced physios and coaches currently estimate these quantities by-eye. Unfortunately, a search showed there is no consensus set of definitions within the physiotherapy and coaching disciplines on how joint angles should be calculated in this manner.

There are several challenges in applying HPE to 3D exercise movement quantification: sufficient key-points are required to compute the joint quantities of interest, models are needed with appropriate training datasets to provide accurate predictions, and an agreed set of metrics is required. To the authors’ knowledge, no method has yet satisfied all of these requirements for general exercise evaluation. Accordingly, this paper tests whether a set of proposed exercise metrics can be inferred directly from single camera view (monocular) video for quantification of exercise quality, over a range of commonly used strength and rehabilitation exercises.

The contribution of this paper is therefore to present a proposed set of pose landmarks for computing frequently used joint angles/metrics of interest to physiotherapists and coaches in assessing common strength-building human exercise movements. The paper then proposes a set of rules for computing these metrics for a range of exercises directly from anatomical key-points, and evaluates the accuracy of these using a 3D human pose model (Strided Transformer) trained with ground truth data derived from VICON motion capture of common rehabilitation exercises.

## 2 Methods

The following components are required: a set of exercises with evaluation metrics, a set of 3D key-points for the body sufficient to compute the chosen exercise evaluation metrics, a model and associated training data to predict the 3D key-points directly from video, a means to compute the metrics from the 3D key-points and ground truth data to evaluate the prediction accuracy. These components are all presented here.

### 2.1 Required exercises and metrics

**Fig. 2:**
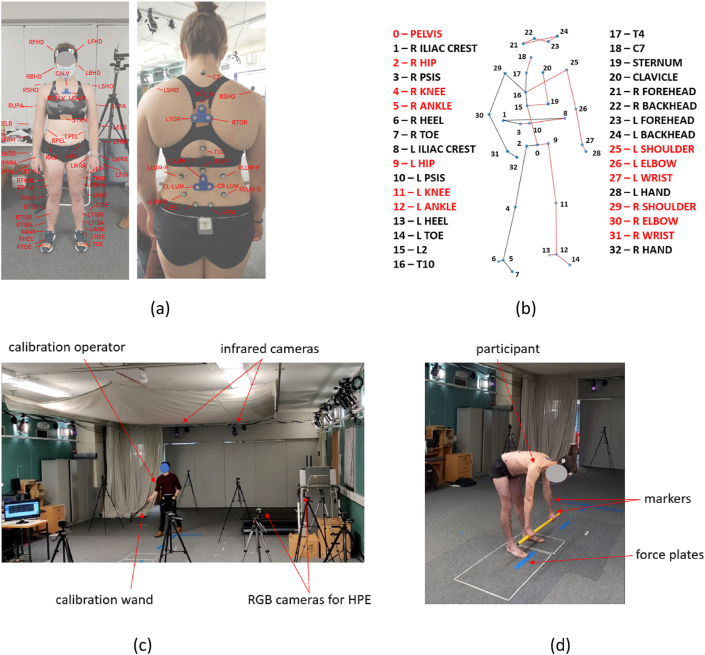
(a) Marker set for ground truth data collection, (b) corresponding thirty-three key-point skeletal model, where black labels correspond to markers, and red labels correspond to joint centres calculated by the VICON plug-in-gait model, (c) VICON ground truth data capture experimental setup with calibration procedure and (d) mobile device camera view of participant completing a deadlift used for human pose estimation (HPE). (For interpretation of the references to colour in this figure legend, the reader is referred to the Web version of this article.)

A pair of professional physios (GF, MB) working at Leinster Rugby created a needs table for general functional movements that included a list of exercises and a set of metrics of interest for each exercise, see Table 1. These metrics were used to determine the anatomical key-points needed for the pose estimation skeleton, see Fig. 2b. Briefly, in addition to the main key-points common to many HPE models (ankles, knees, hips, shoulders, elbows, wrists, head), the toes and pelvis (left and right PSIS and Iliac crest) and the spine (spinous processes of the vertebral bodies L2, T10, T4 and C7) and the sternum and clavicle are included (Fig. 2b).

**Table 1:** Principal functional exercises and evaluation metrics.

	Knee varus/valgus angle (°)	Ankle flexion angle (°)	Knee flexion angle (°)	Hip flexion angle (°)	Trunk angle relative to vertical (°)	Low Spine Flexion (°)	Mid Spine Flexion (°)	Upper Spine Flexion (°)	Rib flare or pelvic tilt	Neck position	Shoulder flexion angle (°)	Symmetry of ASIS height relative to horizontal (°)	Shin angle relative to vertical (°)
*Back squat*	**X**	**X**	**X**	**X**	**X**	**X**	**X**	**X**	**X**	**X**			
*Front squat*	**X**	**X**	**X**	**X**	**X**	**X**	**X**	**X**	**X**	**X**	**X**		
*Overhead squat*	**X**	**X**	**X**	**X**	**X**	**X**	**X**	**X**	**X**	**X**	**X**		
*Single leg squat*	**X**	**X**	**X**	**X**	**X**	**X**	**X**	**X**	**X**	**X**	**X**	**X**	
*Trap bar*	**X**	**X**	**X**	**X**	**X**	**X**	**X**	**X**	**X**	**X**	**X**		
*Deadlift/hip hinge*	**X**	**X**	**X**	**X**	**X**	**X**	**X**	**X**	**X**	**X**	**X**		
*Counter Move – Double*	**X**	**X**	**X**	**X**	**X**								**X**
*Counter Move – Single*	**X**	**X**	**X**	**X**	**X**								**X**
*Drop Jump – Double*	**X**	**X**	**X**	**X**	**X**							**X**	**X**
*Drop Jump – Single*	**X**	**X**	**X**	**X**	**X**								**X**

### 2.2 Ground truth data collection

Following ethical approval from the Trinity College Dublin School of Engineering Research Ethics Committee, a first set of ground truth data was collected using VICON motion capture technology (VICON, Oxford, UK) integrated with force plates, see Fig. 2c. Four female and four male athletes (aged 20–30, from a variety of sporting backgrounds) were recruited via Trinity College Dublin and Leinster Rugby. The standard VICON full body plug-in-gait marker set was augmented with additional markers, and clusters were added to the clavicles and back, see Fig. 2a.

The exercises included three different depth squats (x3 shallow, x3 normal, x3 deep), five deadlifts and five counter movement jumps (CMJ), see Fig. 2d. A second data collection involving four male athletes used a VICON system with six cameras. The data collected from these experiments was processed using VICON Nexus 2.0 software. A combination of joint centres and 3D marker positions was used to build the thirty-three key-point skeleton used in the pose estimation model, see Fig. 2b.

### 2.3 Metrics definition

Single view markerless motion capture accuracy is mostly assessed in the computer vision community using a metric known as Mean Per Joint Position Error (MPJPE). This is a good overall positional metric and we used this for the loss function in the training phase. However, MPJPE has little direct practical significance. Instead, for prediction effectiveness we directly assessed errors in the joint angle metrics set out in Table 1. These metrics address angles of the main articulating joints (ankles, knees, hips, shoulders) and well as the orientation of the pelvis, spinal bending and rib flare). The metrics are measured either from the orientation of a rigid body constructed from at least three key-points or the angle between two segments. For the latter, each segment was formed by two key-points and, when appropriate, the segment was projected onto relevant principal anatomical planes, with definitions presented in Table 2. These definitions were designed to mathematically codify the every-day interpretation of these metrics by physiotherapists so that they can be computed uniquely from the thirty-three key-point pose landmarks (Fig. 2b) extracted from video (Fig. 2d).

**Table 2:** Definition of metrics from key-points and principal anatomical planes.

Metric	Definition	Schematic	Metric	Definition	Schematic
Ankle Flexion	Angle of shin (knee – ankle) and front foot (ankle – toe)		Knee Flexion	Angle of thigh (hip – knee) and shin (knee – ankle)	
Hip Flexion	Angle of lower spine (L2 – hip) and thigh (hip – knee)		Shoulder Flexion	Angle of torso (mid-hips to shoulder) and upper arm (shoulder – elbow)	
Knee Varus/Valgus	Angle of transverse plane normal and shin (knee – ankle, in coronal plane)		Trunk angle to vertical	Angle of transverse plane normal and torso (C7 marker – pelvis (mid hips, in sagittal plane)	
ASIS asymmetry relative to horizonal	Angle of sagittal plane normal and L&R iliac crest markers (in coronal plane)		Shin angle to the vertical	Angle of transverse plane normal and shin (in sagittal plane)	
Upper spine flexion	Angle of vector T10 - T4 and vector T4 - T7		Mid Spine Flexion	Angle of vector L2 - T10 and vector T10 - T4	
Lower Spine Flexion	Angle of midpoint of L&R PSIS-L2 and L2-T10		Rib Flare	1. ‘Torso’ rigid body from sternum, clavicle & T4 Angle torso body vertical C7-pelvis (mid hips) in coronal plane.	
Pelvic Tilt	1. ‘Pelvic’ rigid body from PSIS and iliac crests. Angle of pelvic body vertical and C7-pelvis (mid-hips) in coronal plane.		Neck position	1. ‘Head’ rigid body from all head markers Angle of head vertical and C7-pelvis (mid hips) in coronal plane.	

**Fig. 3:**
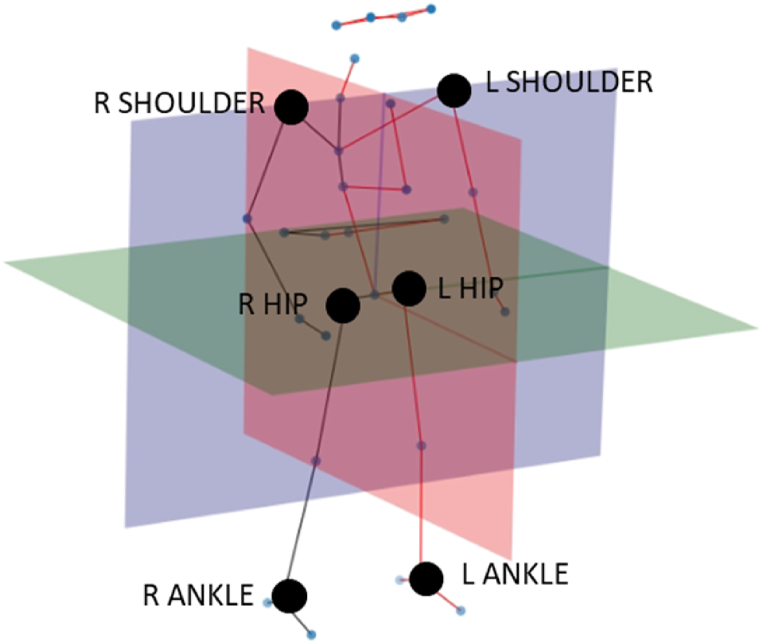
Anatomical planes fitted to pelvic centric pose estimation skeleton as a precursor to joint angle predictions.

As a first step, it was necessary to define the principal anatomical planes. The coronal plane (blue in Fig. 3) was first fitted through the pose key-points defining the shoulders, hips, and ankles using the least squares error method. The sagittal plane (red) was obtained using the normal of the coronal plane and the vector between the C7 spinal marker and the pelvis projected onto the coronal plane (C7-pelvis was defined as the vertical vector of the body). The transverse plane (green) was perpendicular to the vertical vector of the body, found use the cross product.

The individual metrics were then computed using the key-points and the anatomical planes according to the definitions in Table 2. These were defined followed detailed discussions regarding the interpretation of joint angles by physiotherapists. The spine and pelvis was divided into four segments, leading to upper, mid and lower spine flexion.

### 2.4 A proposed 3D pose estimation skeleton with extended key-points

The pose estimation models used in this study were based on Detectron2 [30], a popular 2D key-point detector (Detectron2) and Strided Transformer [31], which “lifts” 2D image key-points to pelvic (mid-hips) centric 3D spatial coordinates. The Strided Transformer model was chosen as it is freely available and because of its temporal components which help to smooth any jittery movement between frames. Since training a 2D pose detector was outside the scope of this work and Detectron2 (and other common 2D key-point detectors) typically only uses 17 key-points (Coco standard [32]), the 3D model was trained to directly lift seventeen 2D key-points to the thirty-three 3D key-points identified in Fig. 2b. This was done using “virtual cameras” to project the known coordinates of the seventeen 3D key-points obtained from the ground truth data into 2D image coordinates for training purposes to represent a range of possible camera positions. The camera intrinsic parameters from the popular Human3.6 M dataset [33] were used, with some modifications, to ensure valid projections. This approach, together with the thirty-three 3D key-points was used for training the 3D pose estimator. In addition to the standard coordinate normalisation that Strided Transformer performs, a fixed sized bounding box was added both in training and inference to capture the height of the person in pixel coordinates.

The models were trained using PyTorch on a single NVIDIA GeForce RTX 3090 GDDR6X V100 GPU with 24 GB of memory. The training dataset consisted only of the ground truth data collected using the motion capture system described above, since open-source datasets have insufficient detail. Because of this, all available VICON data was used for training. The loss function was divided into two components, with 80% of the loss being the MPJPE and 20% being mean squared error (MSE) of the knee angles. Other loss functions were attempted but did not yield improved results.

Following training, validation was performed using the RMSE of each metric for each exercise over all repetitions for all participants. Two thresholds were set to evaluate model performance. If a metric MSE was less than 12°, it was considered ‘good’, as this is better than “by-eye” judgement of physiotherapists [1], and if it was less than 6°, it was considered ‘very good’ is this is less than half of the reported “by-eye” judgement error. The best model was selected based on the number of metrics above each threshold. If there were still multiple model “checkpoints” to choose from, the checkpoint with the most metrics above 6° mean root mean square accuracy was selected.

For validation, the exercises performed during the VICON experiments were recorded with multiple mobile device cameras placed around the athlete in a radius of approximately 3 m, both on the floor and on tripods at approximately 1.5 m height, see Fig. 2c&d. These positions simulate the likely actual positions of handheld cameras used to record exercise movements by physiotherapists and coaches in future. These videos were then used as inputs to the trained pose estimator, and the MSE of each metric over all repetitions and by all participants was computed for each exercise.

## 3 Results

Table 3 shows the root mean square errors (degrees) of the prediction metrics from the pose estimation model assessed against the VICON as ground truth, with metrics defined according to Table 2. “Good” results are shown in light green, “very good” results are shown in dark green and the remainder are shown in grey. Table 3 shows the error over all participants and all repetitions of each exercise by these participants, and the thresholds for colour coding the results (“good” and “very good”) are informed by Ref. [1], who found that physiotherapists could assess joint angles to about 12° accuracy in low-speed functional movements. Table 4 shows the corresponding coefficient of variation of the errors (standard deviation/mean) results for the same metrics. Some metrics have high coefficients of variation, but this is a reflection of the low mean errors. For example, for knee varus/valgus in left single-legged drop jumps, the error is 3° and the standard deviation of the error is also 3°, leading to a coefficient of variation of 1.

**Table 3:**
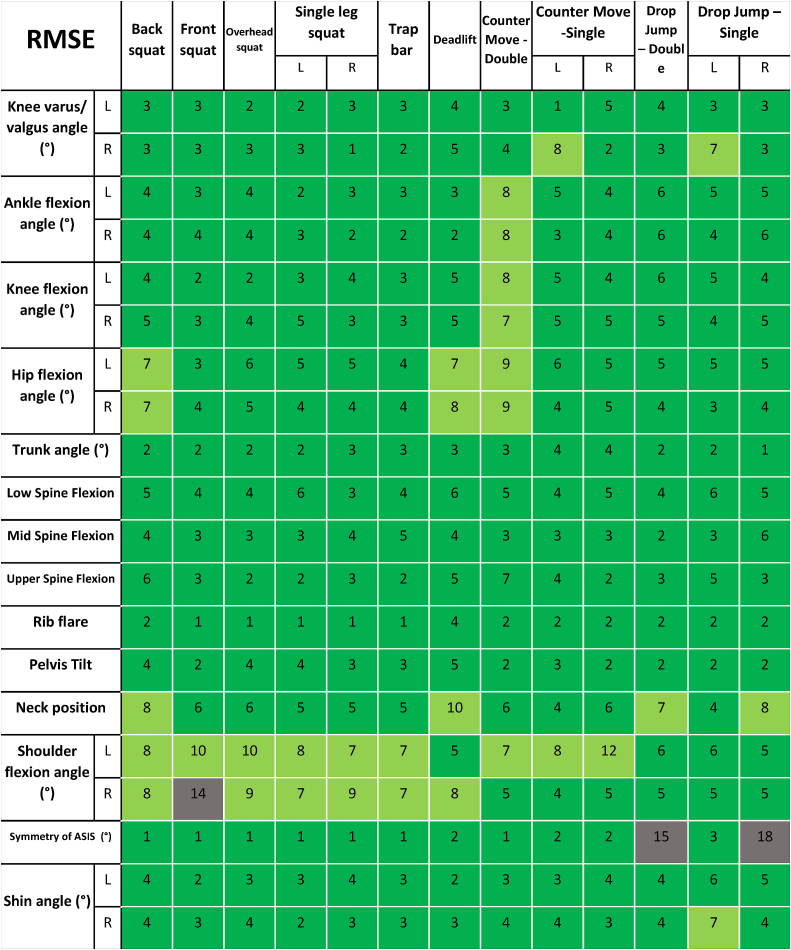
Root mean squared errors (degrees) of the prediction metrics for the thirty-three key-point single camera pose estimation model, with metrics defined according to Table 2. Light green denotes ‘good’ results, dark green denotes ‘very good’ results, grey denotes ‘improvement needed’. Thresholds informed by Ref. [1].

**Table 4:** Coefficient of Variation of the prediction metrics for the thirty-three key-point single camera pose estimation model, with metrics defined according to Table 2.

COV		Back squat	Front squat	Overhead squat	Single leg squat		Trap bar	Deadlift	Counter Move -Double	Counter Move -Single		Drop Jump - Double	Drop Jump - Single	
					L	R				L	R		L	R
Knee varus/valgus angle (**°**)	L	0.81	0.81	0.97	0.95	0.98	0.91	0.4	0.83	1.17	0.76	0.86	1	0.96
	R	0.65	0.77	0.77	0.8	1.18	0.94	0.34	0.51	1.02	0.92	0.99	0.73	0.98
**Ankle flexion angle (°)**	L	0.61	0.74	0.68	1.05	0.78	0.79	0.63	0.53	0.78	0.7	0.74	0.65	0.77
	R	0.65	0.61	0.72	0.61	0.98	0.9	0.94	0.58	0.77	0.76	0.75	0.83	0.73
**Knee flexion angle (°)**	L	0.77	0.96	0.87	0.99	0.81	0.84	0.69	0.57	0.66	0.77	0.74	0.82	0.75
	R	0.69	0.79	0.79	0.62	0.82	0.81	0.65	0.56	0.75	0.7	0.68	0.77	0.67
**Hip flexion angle (°)**	L	0.58	0.73	0.56	0.61	0.64	0.71	0.56	0.51	0.6	0.69	0.62	0.59	0.67
	R	0.59	0.69	0.63	0.75	0.72	0.78	0.54	0.53	0.78	0.74	0.69	0.84	0.68
**Trunk angle (°)**		1.01	1.15	1.19	1.11	0.99	1.04	0.93	0.92	0.79	0.74	1.01	1.23	1.29
**Low Spine Flexion**		0.45	0.67	0.71	0.62	0.77	0.66	0.53	0.47	0.65	0.54	0.73	0.74	0.77
**Mid Spine Flexion**		0.53	0.63	0.69	0.93	0.68	0.55	0.68	0.67	0.8	0.71	0.89	0.84	0.91
**Upper Spine Flexion**		0.32	0.66	0.74	0.84	0.87	0.98	0.42	0.27	0.64	1.04	0.92	1.1	0.85
**Rib flare**		1.06	1.61	2.47	1.49	1.65	2.01	0.77	1.11	1.3	1.32	1.22	1.6	1.29
**Pelvis Tilt**		0.78	1.18	0.8	0.72	0.86	0.93	0.73	0.99	0.89	0.97	0.92	1.05	0.99
**Neck position**		0.47	0.63	0.68	0.71	0.72	0.77	0.54	0.61	0.7	0.58	0.59	0.76	0.46
**Shoulder flexion angle (°)**	L	0.36	0.24	0.25	0.47	0.44	0.35	0.55	0.43	0.41	0.24	0.56	0.7	0.62
	R	0.38	0.18	0.28	0.55	0.45	0.39	0.4	0.55	0.63	0.55	0.7	0.72	0.7
**Symmetry of ASIS (°)**		1	1.41	1.65	1.83	1.5	1.65	1.85	1.13	1.15	1.03	1.15	0.63	0.97
**Shin angle (°)**	L	0.83	0.92	1.01	0.89	0.79	0.99	0.82	0.92	0.89	0.73	0.65	0.73	0.7
	R	0.8	0.87	0.79	1.09	0.96	0.94	0.57	0.89	0.76	0.94	0.76	0.55	0.69

## 4 Discussion and implications

There are three main contributions to this study: the choice of body key-points, the definition of the exercise metrics and the evaluation of the accuracy of a pose estimation model using monocular video as input.

We have proposed a novel set of thirty-three human pose landmark key-points (Fig. 2b) necessary and sufficient for computing joint angles of frequent interest to physiotherapists and coaches in assessing common strength-building human exercise movements (Table 1). Current open-source models (eg Meta's VideoPose3D [12]) have reduced key-point models which do not allow computation of some of these metrics (Fig. 1). Accordingly, the proposed detailed pose configuration with 33 key-points is useful for sports biomechanics applications where biomechanically meaningful and readily interpretable movement analysis is needed from single camera view video analysis, and where the accuracy and complexity of the ISB 2002 joint angle definitions [29] is not required, and the necessary time investment is not desirable.

Further, a set of defined metrics for common exercise classification was developed (Table 2), and their mathematical meaning is set out in Table 2. These definitions are derived from a biomechanical interpretation of joint angle calculations as they would be performed by a physiotherapist making a “by-eye” judgement. While acknowledging that these definitions are not universal, they are unambiguous and mathematically unique and hence repeatable, and the definitions could be revisited in future. Their definition satisfies an important requirement for potential application of human pose estimation applied to assessing the consistency of human movement in exercise quantification. This is particularly important in physiotherapy and coaching where progression of joint angle changes between repetitions or over a longer time-period is generally more important than precise measurements of joint angles, particularly given the limited ability of physiotherapists to accurately quantify joint angles by eye [1].

Finally, we have shown that the predictive capacity of a current 3D pose estimation model (Strided Transformer [31]) is quite high when trained on a novel VICON motion capture dataset of functional exercise movements, and is therefore useful in assessing the proposed physiotherapy prediction metrics. Table 3 shows only 1% of metrics have mean predictions poorer than typical physiotherapist “by-eye judgements” (12° threshold from Ref. [1]). The root mean square angle errors in Table 3 were within 10° for all exercises for the following metrics: shin angle, knee varus/valgus and left/right flexion, hip flexion and pelvic tilt, trunk angle, spinal flexion lower/upper/mid and rib flare. Larger errors (though still all within 15°) were observed for shoulder flexion and ASIS asymmetry in some exercises, notably front squats and drop-jumps, though further training data may improve these predictions. The ability to reconstruct these metrics from monocular video reflects the versatility of the Strided Transformer model, the power of the virtual cameras during training and the directly applicable nature of the training data. By comparison, a recent two camera method found mean absolute errors (MAE) for joint rotations ranging from 2° to 10° for squat, sit-to-stand and drop jumps, partly reflecting the accuracy benefits of inclusion of a second camera (but which requires a calibration step), and also the potential for error averaging when assessing errors with MAE.

There are several limitations to this work. The findings presented provide provisional support for the hypothesis that a set of exercise metrics can be inferred from single camera view video for quantification of exercise quality. However, further testing of this approach in a real-world setting is needed to test the effects of lighting level, clothing and camera positioning on joint angle predictions. Further, potential future application to exercises or movements where, for example, the elbow angles and hand positions are of importance will require additional training and testing. In the future, application of recent novel optimization strategies (such as [[34], [35], [36], [37], [38], [39]]) from related areas will likely further improve the quality of human pose prediction from video.

The work presented here relates to a subset of exercises of interest to physiotherapists and coaches, and these exercises feature less self-occlusion than (for example) press-ups. Due to the logistics of marker-based motion capture, the cohort of participants for the training dataset is small, and should be expanded in future. Specifically, it is likely that additional benefits would accrue from expanding the training dataset to include a more diverse population of athletes with varying body shapes and clothing and with a wider range of environmental conditions and exercises, the latter including those with higher degrees of self-occlusion.

Furthermore, in future improved accuracy may be achieved either through improvements in 2D key point estimation, better definition of camera recording angles, and improved models and training methods for lifting 2D key point pixel positions to spatial coordinates.

The importance of the appropriate model training dataset is clear in this work, since existing movement datasets such as the popular Human3.6 M [33] do not contain sufficiently similar movements to the functional exercises under consideration here, and even physiotherapy specific datasets like UIPRMD [40] were not captured with sufficient marker information to allow the full thirty-three joint skeleton reconstruction presented here to be adequately trained. Further, the generally high accuracy of the predictions for these exercises (Table 3) is a result of model training with ground truth data containing these same exercises and generalization needs to be considered in future. Further improvements in accuracy may also be achieved through sensor fusion, for example by combining with wearable IMUs. However, this requires consideration of the trade-off between ease-of-use and accuracy. Depth cameras are also a possibility.

This paper addresses the exercises and metrics in Table 1. These are a common set of exercises present in many strength-building programmes across a variety of sports. These exercises and metrics could be expanded in the future, and the human key-point “skeleton” can be further developed as required according to these needs. Similarly, while the focus here has been on joint angle reconstruction for sports physiotherapy applications, a similar approach is likely to be fruitful in other applications such as at-home physiotherapy, ergonomics and sporting skills acquisition. The key components remain the mathematical definition of the required metrics from human body key-points, a sufficiently detailed set of human pose key-points and appropriate training data to train a pose estimation model. Provisional findings show that generalization of model predictions to other exercises such as single deadlifts and golfing movements may be achieved given suitable training data, but otherwise prediction accuracy reduces quickly for non-validated movements.

There are a number of implications of this work. The first is that pose estimation models have the potential to infer a wide range of exercise joint angle metrics which are at least as accurate as an assessment by a trained physiotherapist. A particular benefit would accrue in an environment where a physiotherapist or a coach assesses many athletes over several time points, and where automated recording of metrics associated with different exercises would be very useful to understand patterns of general athletic development and return from injury pathways. This approach may also be useful for at-home physiotherapy and personal training. In principle, the approach will likely also work for a broader range of exercises than those considered in this paper. The theoretical accuracy will depend on the degree of occlusion present in performing the exercise, and the availability of training data for those exercises.

Another implication is that generic pose estimation models (eg Ref. [12]) with insufficient detail in key anatomical areas and not trained on specific exercise data are unlikely to yield results with sufficient accuracy for practical application for physiotherapists and coaches. Conversely, when suitable training data is available and the skeletal model is of sufficient detail, the potential is there for quick and accurate tools with a wide range of applications. In future, a real-time or semi-real-time implementation of this approach could be used to provide quantitative feedback on exercise performance to athletes and their physiotherapists and coaches. Tracking changes in exercise execution over time with this approach could be used to facilitate adjustments to rehabilitation and training programmes, potentially reducing injury risk and increasing training efficacy.

## 5 Conclusions

We have proposed a set of thirty-three human pose landmarks sufficient for computing frequently used joint angles of interest to physiotherapists and coaches in assessing common strength-building human exercise movements. Further, we have defined unique mathematical definitions for computing metrics for these exercises from the proposed thirty-three key-points, designed to interpret the qualitative definitions applied by physiotherapists in by-eye assessments. We found good accuracy of the Strided Transformer 3D pose model in predicting these metrics from a single mobile device camera when trained on a suitable set of functional exercises recorded using a VICON motion capture system as ground truth data. The root mean square angle errors were within 10° for all exercises for the following metrics: shin angle, knee varus/valgus and left/right flexion, hip flexion and pelvic tilt, trunk angle, spinal flexion lower/upper/mid and rib flare. Larger errors (though still all within 15°) were observed for shoulder flexion and ASIS asymmetry in some exercises, notably front squats and drop-jumps. The metrics and the pipeline proposed here provide a practical means to assess quality of exercise performance using single camera view video. This provides a potential means to move exercise assessment from a largely qualitative basis to a quantitative basis.

## Funding Statement

The authors are grateful to 10.13039/501100001588Enterprise Ireland and 10.13039/501100001602Science Foundation Ireland for financial support. These sponsors had no role in the study design, nor any involvement in the submission of this paper for publication.

## Ethical Statement

This research has been approved on Sep 1st, 2021 from the Faculty of School of.

Computer Science and Statistics Research Ethics Committee (application number (20201210), Trinity College Dublin (e-mail: ethicscommittee@tcd.ie).and all the subjects gave written informed consent to participate and to publish the data.

## Data availability Statement

The data associated with this study is not available in a public repository because the authors do not have permission to share the data.

## CRediT authorship contribution statement

**Clara Mercadal-Baudart:** Writing – original draft, Visualization, Validation, Methodology. **Chao-Jung Liu:** Writing – review & editing, Software, Methodology, Formal analysis. **Garreth Farrell:** Writing – review & editing, Resources, Conceptualization. **Molly Boyne:** Writing – review & editing, Resources, Methodology. **Jorge González Escribano:** Writing – review & editing, Software, Methodology, Formal analysis. **Aljosa Smolic:** Writing – review & editing, Supervision, Software, Resources, Investigation, Conceptualization. **Ciaran Simms:** Writing – original draft, Supervision, Methodology, Funding acquisition.

## Declaration of generative AI and AI-assisted technologies in the writing process

During the preparation of this work the authors used ChatGPT in order to improve language and wording of the authors who are non-native English speakers. After using this tool/service, the authors reviewed and edited the content as needed and take full responsibility for the content of the publication.

## Declaration of competing interest

The authors declare that they have no known competing financial interests or personal relationships that could have appeared to influence the work reported in this paper.

## Appendix Metrics Definitions

This appendix is a companion to Table 2, providing a brief rationale to the metric definitions used. Angles between segments are generally computed using the inverse cosine of the dot product of unit vectors defining the segments. In all cases, in the absence of consensus amongst physiotherapists and coaches for the definition joint angles, these definitions are informed by our mathematical interpretation of the angles as they are used in everyday practice.

**Ankle Flexion:** this is the angle between the foot segment (defined by the ankle and toe markers) and the shin segment (defined by the ankle and knee markers). The ankle flexion angle in quiet standing is defined as 0°. Plantarflexion is positive.

**Knee Flexion:** this is the angle between the shin segment (defined by the ankle and knee markers) and the thigh segment (defined by the knee and hip markers). The knee flexion angle in quiet standing is defined as 0°.

**Hip Flexion:** this is the angle between the thigh segment (defined by the knee and hip markers) and the pelvis segment (defined here by the L2 lumbar vertebra and hip markers). The hip flexion angle in quiet standing is defined as 0°.

**Shoulder Flexion:** this is the angle between the torso segment (defined by the mid-hip marker and shoulder marker) and the upper arm segment (defined shoulder and elbow markers). The shoulder flexion angle in quiet standing is defined as 0°.

**Knee Varus/Valgus:** this is the angle between the normal to the transverse plane (see main paper for definition) and the shin segment (defined by the ankle and knee markers).

**Trunk angle to vertical:** this is the angle between the normal to the transverse plane (see main paper for definition) and the torso segment (defined by the mid-hip and C7 cervical vertebra marker). The trunk angle to vertical in quiet standing is defined as 0°.

**ASIS asymmetry relative to vertical:** this is the angle between the normal to the sagittal plane (see main paper for definition) and the pelvis segment (defined here by the left and right iliac drest markers).

**Shin angle to vertical:** this is the angle between the normal to the transverse plane (see main paper for definition) and the shin segment (defined by the ankle and knee markers).

**Upper spine flexion:** this is the angle between the vector defined by thoracic vertebra T10 and T4, and the vector defined by thoracic vertebra T4 and T7. The upper spine angle in quiet standing is defined as 0°.

**Mid spine flexion:** this is the angle between the vector defined by lumbar vertebra L2 and thoracic vertebra T10, and the vector defined by thoracic vertebra T10 and T4. The mid spine angle in quiet standing is defined as 0°.

**Lower spine flexion:** this is the angle between the vector defined by the midpoint of left and right PSIS and lumbar vertebra L2, and the vector defined by lumbar vertebra L2 and thoracic vertebra T10. The lower spine angle in quiet standing is defined as 0°.

**Rib flare:** a “torso” rigid body is defined by the three markers sternum, clavicle and T4. Rib flare is the angle between the torso normal and the vector from cervical vertebra C7 to the mid-hips marker. The rib flare in quiet standing is defined as 0°.

**Pelvic tilt:** a “pelvis” rigid body is defined by the four markers Iliac crest and PSIS (left and right). Pelvic tilt is the angle between the pelvis normal and the vector from cervical vertebra C7 to the mid-hips marker. The pelvic tilt in quiet standing is defined as 0°.

**Neck Position:** a “head” rigid body is defined by all the head markers. Neck position is the angle between the head vertical and the vector from cervical vertebra C7 to the mid-hips marker projected onto the coronal plane. The neck position in quiet standing is defined as 0°.

## References

[1] Abbott E. (2022). Physiotherapists could detect changes of 12 degrees or more in single-plane movement when observing forward bending, squat or hand-over-head: a cross-sectional experiment. Musculoskeletal Science and Practice.

[2] Apta (2019).

[3] Skjaerven L., Kristofferson K., Gard G. (2008). An eye for movement quality: a phenomenological study of movement quality reflecting a group of physiotherapists' understanding of the phenomenon. Physiother. Theory Pract..

[4] Whatman C., Hume P., Hing W. (2013). The reliability and validity of physiotherapist visual rating of dynamic pelvis and knee alignment in young athletes. Phys. Ther. Sport.

[5] Hensley C.P. (2020). Video-based motion analysis Use: a National Survey of Orthopedic physical therapists. Phys. Ther..

[6] Kuster R.P. (2016). Accuracy of KinectOne to quantify kinematics of the upper body. Gait Posture.

[7] Tulipani L. (2018). Validation of an inertial sensor system for physical therapists to quantify movement coordination during functional tasks. J. Appl. Biomech..

[8] Bolink S.A.A.N. (2016). Validity of an inertial measurement unit to assess pelvic orientation angles during gait, sit-stand transfers and step-up transfers: comparison with an optoelectronic motion capture system. Med. Eng. Phys..

[9] Hughes T. (2019). Are tibial angles measured with inertial sensors useful surrogates for frontal plane projection angles measured using 2-dimensional video analysis during single leg squat tasks? A reliability and agreement study in elite football (soccer) players. J. Electromyogr. Kinesiol..

[10] Aoyagi Y. (2022). Development of smartphone application for markerless three-dimensional motion capture based on deep learning model. Sensors.

[11] Bazarevsky V. (2020). BlazePose: on-device real-time body pose tracking. CPVR.

[12] Pavllo D. (2019). 2019 Ieee/Cvf Conference on Computer Vision and Pattern Recognition (Cvpr 2019).

[13] Debnath B. (2022). A review of computer vision-based approaches for physical rehabilitation and assessment. Multimed. Syst..

[14] Haberkamp L.D., Garcia M.C., Bazett-Jones D.M. (2022). Validity of an artificial intelligence, human pose estimation model for measuring single-leg squat kinematics. J. Biomech..

[15] Komatireddy R. (2014). Quality and quantity of rehabilitation exercises Delivered by A 3-D motion Controlled camera: a Pilot study. Int J Phys Med Rehabil.

[16] Leightley D., McPhee J.S., Yap M.H. (2017). Automated analysis and quantification of human Mobility using a depth sensor. IEEE J Biomed Health Inform.

[17] Cao Z. (2021). OpenPose: Realtime Multi-person 2D pose estimation using Part Affinity Fields. IEEE Trans. Pattern Anal. Mach. Intell..

[18] Chang J.Y., Moon G., PoseLifter K.M. Lee (2020). Absolute 3D human pose lifting network from a single noisy 2D human pose. CVPR.

[19] Arrowsmith C. (2022). Physiotherapy exercise classification with single-camera pose detection and Machine learning. Sensors.

[20] Uhlrich S. (2023). OpenCap: human movement dynamics from smartphone videos. PLoS Comput. Biol..

[21] Goncharow P.N., Beaudette S.M. (2022). Assessing time-varying lumbar flexion-Extension kinematics using automated pose estimation. J. Appl. Biomech..

[22] Cunha A.B. (2020). Assessing the validity and reliability of a new video Goniometer App for measuring joint angles in Adults and Children. Arch. Phys. Med. Rehabil..

[23] Bittner M. (2023). Towards single camera human 3D-kinematics. Sensors.

[24] Hisham M.N. (2022). Mono camera-based human skeletal tracking for squat exercise Abnormality detection using double Exponential smoothing. Int. J. Adv. Comput. Sci. Appl..

[25] Vakanski A., Ferguson J.M., Lee S. (2017). Metrics for performance evaluation of Patient exercises during physical Therapy. Int J Phys Med Rehabil.

[26] Liao Y., Vakanski A., Xian M. (2020). A deep learning framework for assessing physical rehabilitation exercises. IEEE Trans. Neural Syst. Rehabil. Eng..

[27] Liao Y. (2020). A review of computational approaches for evaluation of rehabilitation exercises. Comput. Biol. Med..

[28] Wang J. (2020).

[29] ISB (2002). ISB recommendation on definitions of joint coordinate system of various joints for the reporting of human joint motion—part 1: ankle, hip, and spine. J. Biomech..

[30] Wu Y., Kirillov A. (2019). Detectron2..

[31] Li W. (2022).

[32] Lin T. (2014). Computer Vision and Pattern Recognition.

[33] Ionescu C. (2014). Human3.6m: Large scale datasets and predictive methods for 3d human sensing in natural environments. IEEE Trans. Pattern Anal. Mach. Intell..

[34] Ozcelik Y., Altan A. (2023). Overcoming Nonlinear dynamics in Diabetic RetinopathyClassification: a Robust AI-based model with Chaotic SwarmIntelligence optimization and Recurrent LongShort-Term Memory. MDPI fractal and fractional.

[35] Sezer A., Altan A. (2021). Detection of solder paste defects with an optimization-based deep learning model using image processing techniques. Solder. Surf. Mt. Technol..

[36] Ezugwu A. (2022). Prairie Dog optimization algorithm. Neural Comput. Appl..

[37] Zare M. (2023). A Global best-guided Firefly algorithm for engineering Problems. Jounral of Bionic Engineering.

[38] Hu G. (2023). Genghis Khan shark optimizer: a novel nature-inspired algorithm for engineering optimization. Adv. Eng. Inf..

[39] Hu G. (2023). DETDO: an adaptive hybrid dandelion optimizer for engineering optimization. Adv. Eng. Inf..

[40] Liao Y., Vakanski A., Xian M. (2019). A deep learning framework for assessing physical rehabilitation exercises. IEEE Trans. Neural Syst. Rehabil. Eng..

